# Mimivirus transcription and translation occur at well-defined locations within amoeba host cells

**DOI:** 10.1128/jvi.00554-25

**Published:** 2025-06-13

**Authors:** Lotte Mayer, Georgi Nikolov, Martin Kunert, Matthias Horn, Anouk Willemsen

**Affiliations:** 1 Centre for Microbiology and Environmental Systems Science, Division of Microbial Ecology, University of Vienna 115351, Vienna, Austria; Michigan State University, East Lansing, Michigan, USA

**Keywords:** giant virus, cytoplasmic replication, viral factory, viral replication cycle, smFISH

## Abstract

Many giant viruses replicate in the cytoplasm in viral factories. How exactly these viral factories are established and where the different steps of the replication cycle occur remain largely obscure. We have developed a single-molecule messenger RNA fluorescence *in situ* hybridization (smFISH) protocol for giant viruses in an *Acanthamoeba* host. Combined with other labeling techniques (FUNCAT, DiD, rRNA FISH, and DAPI), we show the Mimivirus transcription and translation sites during an infection cycle in the amoeba host cell. Although viral mRNA localization changes depend on the infection stage, transcription occurs at well-defined spots within the viral factory. The original viral cores released within the cytoplasm most likely define these spots. When transported outside of the viral factory, the translation of viral mRNA takes place in a well-defined ring surrounding it. With this study, we obtained novel insights into giant virus replication, of which the methods are widely applicable to other viruses for the visualization and quantification of RNA molecules.

**IMPORTANCE:**

Giant viruses have massive particle and genome sizes, which are known to infect unicellular eukaryotes. Although most viruses replicate in the host cell’s nucleus, the giant Mimivirus replicates in viral factories established in the host cell’s cytoplasm. Before this study, the location of the various steps in the Mimivirus replication cycle was largely unknown. By developing new protocols to label giant virus mRNA, protein synthesis, host cell membranes and rRNA, we demonstrate that Mimivirus transcription occurs at well-defined sites within the viral factory. In contrast, translation takes place directly outside of it. This is different from other viruses known to have a cytoplasmic life cycle. These results bring us a step closer to understanding how the genome complexity of viruses influences the virus-host interactions and viral replication strategies.

## INTRODUCTION

Most DNA viruses carry out their replication and transcription either entirely or partially within host nuclei. Although the nuclear environment can considerably enhance the efficiency of replication and transcription (1), it also presents several hurdles, as viral molecules need to be transported to and into the nucleus. Large DNA viruses are particularly affected by hurdles associated with genome trafficking, as the mobility of DNA molecules in the cytoplasm and the nucleus is size-dependent (2), with the main barrier for larger molecules being the cytoskeleton of the host cells (3).

Large DNA viruses belonging to the phylum *Nucleocytoviricota* were previously referred to as nucleocytoplasmic large DNA viruses (NCLDVs). As the name suggests, these viruses can replicate in the host cell nucleus and/or cytoplasm (4). Before the discovery of giant viruses in 2003 (5, 6), the only other large DNA viruses that were known to have an entire cytoplasmic infection cycle were the poxviruses (7, 8). The cytoplasmic life cycle of poxviruses can be roughly divided into virion entry, early transcription, DNA replication, followed by virus assembly and release. It has been recently shown that the giant Mimivirus displays extensive physiologic similarities with poxviruses (9, 10). The Mimivirus genome is not delivered to the host nucleus and never crosses the nuclear membrane (9). A typical Mimivirus infection starts with uptake by the amoeba host cell through phagocytosis. Within the phagosome, the capsid of Mimivirus is opened through a stargate, which allows for the fusion of the viral and the phagosome membrane, forming a large tube (11). Through this membrane tube, the genome core is released (possibly within a vesicle) into the host cytoplasm. Shortly after this delivery, transcription is initiated within the Mimivirus core, followed by a burst of DNA replication within a cytoplasmic viral factory surrounding the core (9). DNA packaging into preformed Mimivirus procapsids proceeds through a portal that is transiently formed at a site distal to the stargate (11). Therefore, despite their distant phylogenetic positions (12), poxviruses and Mimivirus appear to have evolved similar mechanisms to cope effectively with the exit and entry of large genomes.

Due to the large particle and viral factory sizes of most giant viruses (e.g.*,* mimiviruses and marseilleviruses), we can observe infections in single host cells using general nucleic acid staining (e.g.*,* DAPI) combined with fluorescence microscopy. However, this method is not virus-specific and is limited to those giant viruses that form viral factories (e.g.*,* medusaviruses and pandoraviruses do not form viral factories). In this study, we have developed a single-molecule messenger RNA fluorescence *in situ* hybridization (smFISH) protocol that enables the study of active giant virus infections independent of their replication strategy. Combined with other techniques to label DNA, proteins, and membranes, rRNA FISH of the host, and digital PCR, we learn more about the viral transcription and translation sites and demonstrate that the methods presented here have ample applications for studying giant virus infections within amoeba host cells.

## RESULTS AND DISCUSSION

### Setting up smFISH for giant viruses

**Fig 1:**
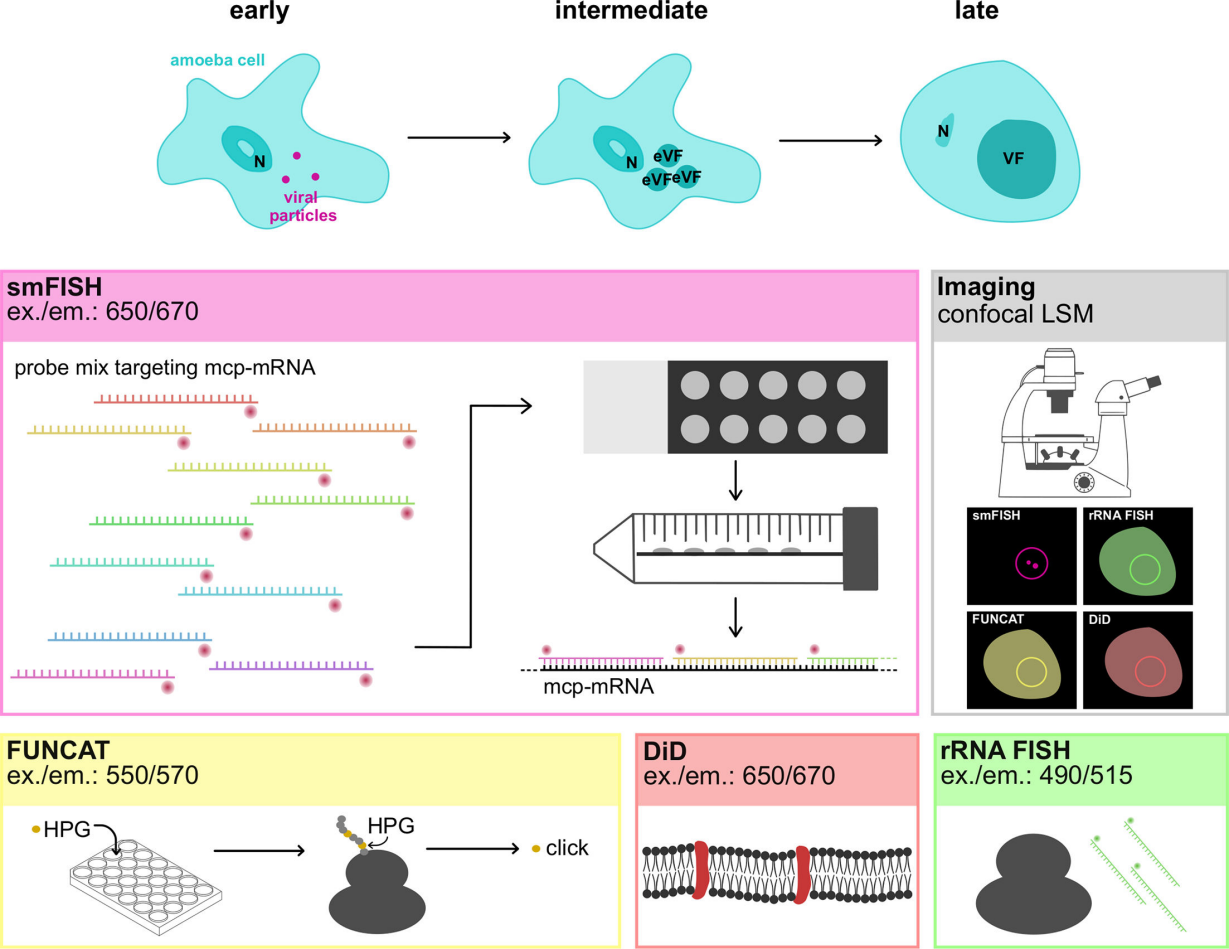
An overview of the methods used in this study. The top of the image shows different infection stages of Mimivirus within an amoeba cell, where the cell gets infected (early: <3 h.p.i), where early viral factories (eVFs) are formed within the cell (intermediate: >3 h.p.i and <6 h.p.i), and where the eVFs have fused into one mature VF (late: >6 h.p.i). To study these infection stages, we use smFISH, fluorescent noncanonical amino acid tagging (FUNCAT), DiD (1,1′-dioctadecyl-3,3,3′,3′- tetramethylindodicarbocyanine, 4-chlorobenzenesulfonate salt) labeling, and ribosomal RNA (rRNA) FISH. The fluorescence of these labels is subsequently visualized using confocal laser scanning microscopy (CLSM).

The single-molecule messenger RNA FISH technique identifies a single mRNA based on the binding of multiple small probes targeted to different locations on the mRNA of interest (13, 14). To set up smFISH for giant viruses within *Acanthamoeba* host cells (Fig. 1), we designed and synthesized a set of 35 individually fluorophore-labeled probes that target the major capsid protein (*mcp*) gene of Mimivirus (Materials and Methods, Table S1). The *mcp* gene is expressed at the late stage of a typical Mimivirus infection, between 6 and 12 h post-infection (h.p.i) (15). In contrast, no *mcp* expression was detected during the early (< 3 h.p.i) and intermediate (> 3 h.p.i and <6 h.p.i) stages of infection (15). Different conditions for fixation and permeabilization of the amoeba cells, hybridization of the probes, washing temperature, and concentration of the probes were tested (Materials and Methods) to obtain the best signal with the optimal conditions in Table 1.

**TABLE 1:** Optimal conditions for smFISH targeting the *mcp* gene of Mimivirus

Step in protocol	Concentration and/or condition used
Sample preparation	
Fixation	4% formaldehyde, 10 min, RT
Permeabilization	70% ethanol, 1 h, 4°C
Hybridization	
Ingredients (final concentration)	4.375 ng/µL probe mix
	20% formamide
	1 mg/mL *E. coli* tRNA
	2× saline-sodium citrate
	0.2 mg/mL bovine serum albumin
	2 mM ribonucleoside-vanadyl complex
	100 mg/mL dextran sulfate
	Nuclease-free water
Temperature	37°C
Time	Overnight
Post-hybridization	
Initial wash	20% formamide, 2× saline-sodium citrate, 15 min 39°C
Final wash	Ice-cold Milli-Q water

Different percentages of formamide, ranging from 0% to 50%, were tested in the hybridization buffer. These formamide series show that although 10% formamide already gives a crisp signal (Fig. S1), the optimal formamide concentration for this probe mix and target sequence is 20%, as this concentration leads to less variability in fluorescent signals between experiments. As a negative control, the cells were treated with RNase A before fixation. No signal was observed when performing the same protocol on RNase-treated cells (Fig. S2).

### Mimivirus *mcp* mRNA localization changes during the infection cycle

By combining electron tomography and fluorescence microscopy, it has been previously shown that each Mimivirus core delivered to the host cytoplasm forms a replication factory and that multiple early viral factories (eVFs) within one host cell can fuse to become one mature viral factory (VF) at late infection stages (9). By using bromodeoxyuridine (BrdU) labeling, it was shown that Mimivirus DNA replication occurs in the host cytoplasm after the exit of the Mimivirus genomes from the viral cores (9). The same study suggests that early Mimivirus transcription is initiated in the cores during DNA release, and bromouridine (BrU) labeling shows that the newly synthesized mRNAs accumulate at discrete cytoplasmic sites that are adjacent to the DNA replication sites (9). To better understand the intracellular localization of Mimivirus *mcp* mRNA during infection, we use the newly developed smFISH method.

**Fig 2:**
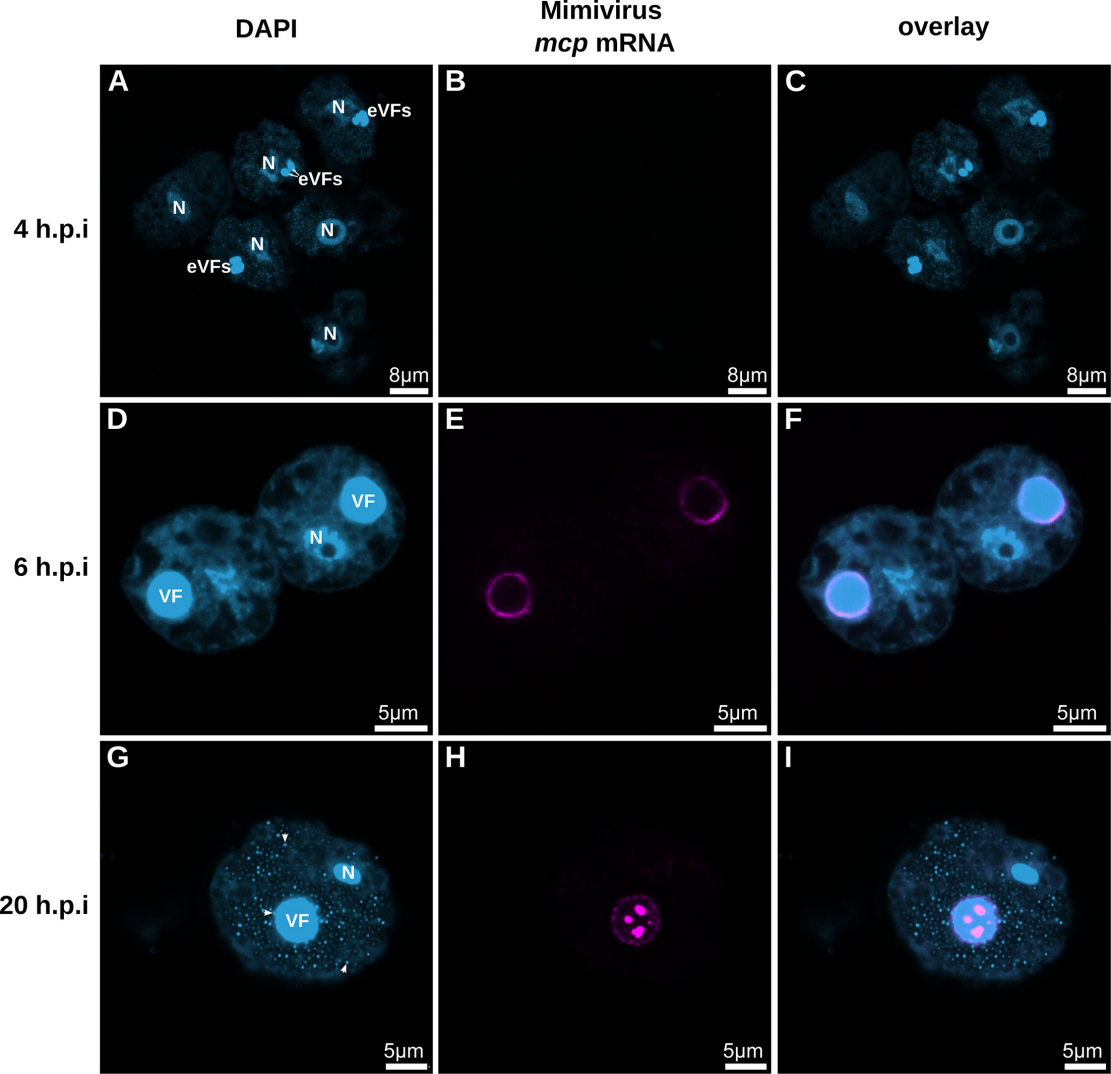
Mimivirus *mcp* mRNA localizes at different sites during the course of infection. (**A, B, C**) Cells were infected with an MOI of 0.5 and harvested at 4 h.p.i. (**A**) DAPI staining (light blue) of six amoeba cells, of which three are infected. A brighter DAPI signal can be observed in the nucleus (N) and the early viral factories (eVFs) of the cells. (**B**) No Mimivirus *mcp* mRNA signal (magenta) is visible at 4 h.p.i. (**C, F, I**) Overlay of DAPI staining (light blue) and Mimivirus *mcp* mRNA signal (magenta). (**D, E, F**) Cells were infected with an MOI of 0.5 and harvested at 6 h.p.i. (**D**) DAPI staining (light blue) of two infected amoeba cells. (**E**) Mimivirus *mcp* mRNA signal (magenta) is localized around the viral factories. (**G, H, I**) Cells were infected with an MOI of 1 and harvested at 20 h.p.i. (**G**) DAPI staining (light blue) of one infected amoeba cell. A brighter DAPI signal can be observed in the nucleus (N), the viral factory (VF), and the viral particles (white arrows indicate three examples) within the cell. The viral particles can be observed budding out of the viral factory and within the rest of the host cell. (**H**) Mimivirus *mcp* mRNA signal (magenta) of one infected amoeba cell at 20 h.p.i. is localized surrounding and at specific spots within the viral factory. The scale bars are drawn on top of the original ones for visibility.

**Fig 3:**
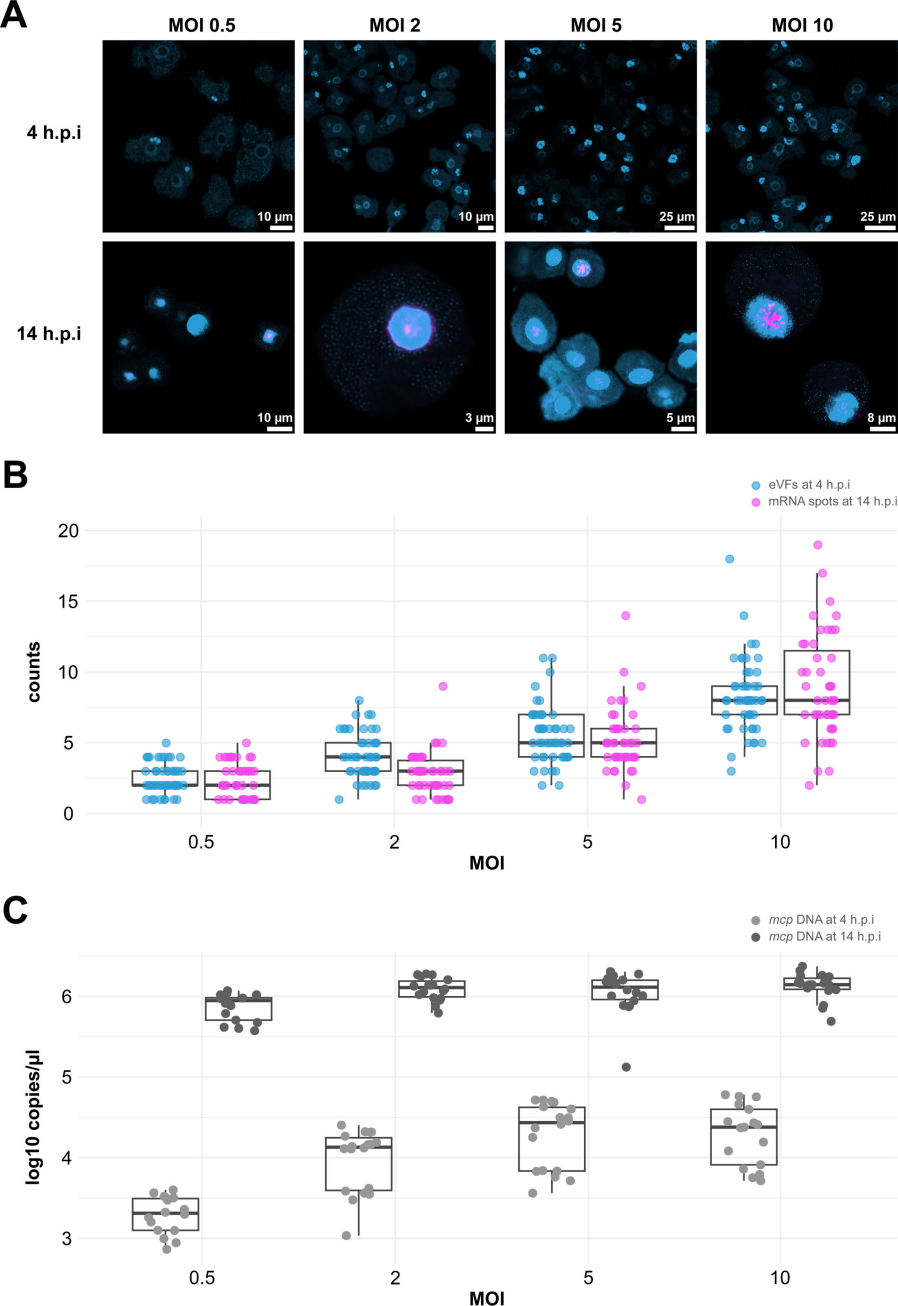
The original viral cores possibly serve separate transcription sites within the fused mature viral factory. (**A**) DAPI staining (light blue) and Mimivirus *mcp* mRNA signal (magenta) of amoeba cells infected with different MOIs at 4 and 14 h.p.i. The Mimivirus *mcp* mRNA signal cannot be observed at 4 h.p.i as expression levels are undetectable at this time point. (**B**) The number of early viral factories counted at 4 h.p.i, and the number of distinct mRNA sites counted at 14 h.p.i after infection with different MOIs. Please note that only infected cells were considered for counting eVFs and mRNA spots. Therefore, the MOI 0.5 and MOI 2 appear inflated compared to the actual MOI used. (**C**) The viral copy number was measured by digital PCR at 4 h.p.i and 14 h.p.i.

**Fig 4:**
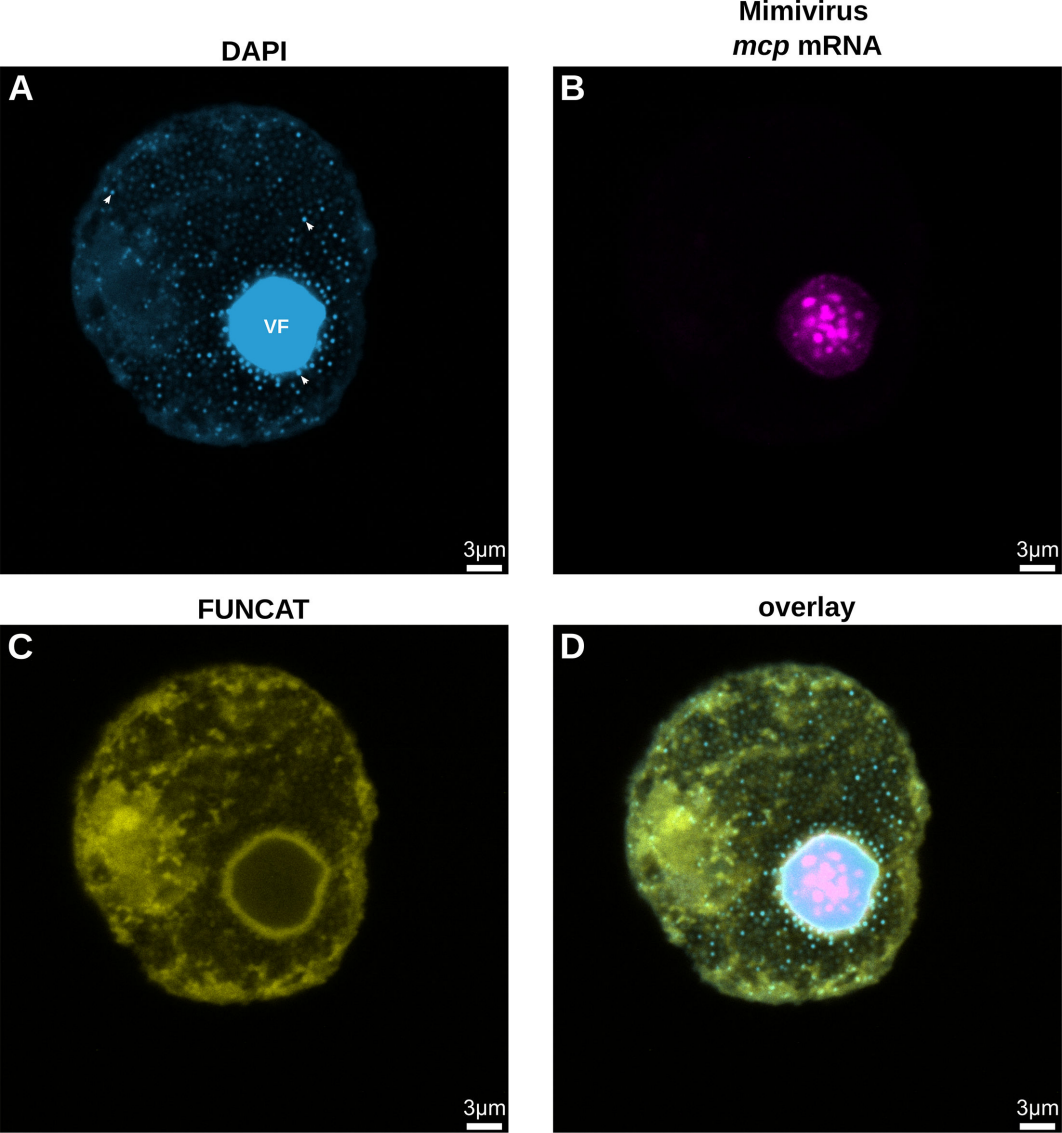
Translation localization coincides with mRNA localization surrounding the viral factory at late infection stages. Cells were infected with an MOI of 1 and harvested at 21 h.p.i. (**A**) DAPI staining (light blue) of one infected amoeba cell. A brighter DAPI signal can be observed in the viral factory (VF) of the cell and for viral particles (white arrows indicate three examples) that can be seen budding out of the viral factory and filling the rest of the cell. (**B**) Mimivirus *mcp* mRNA signal (magenta) of the same cell as in panel A. The mRNA is localized surrounding the viral factories and at specific spots within the viral factories. (**C**) FUNCAT signal (yellow) of the same cell as in panel A. The strong FUNCAT signal suggests that the exported Mimivirus mRNA is translated here. (**D**) Overlay of DAPI staining (light blue), Mimivirus *mcp* mRNA signal (magenta), and FUNCAT (yellow) of the same cell as in panel A. For visibility, the scale bars are drawn on top of the original ones.

*Acanthamoeba terricola* Neff host cells were infected with Mimivirus, and the cells were harvested at different time points during the infection cycle. At the intermediate infection stage of 4 h.p.i, we observe multiple eVFs within each host cell that later fuse (> 6 h.p.i) to become mature VFs (Fig. 2; Fig. S3 and S4). Consistent with transcriptomic data (15), we do not observe Mimivirus *mcp* mRNA at 4 h.p.i (Fig. 2B and 3A; Fig. S3). The earliest time point where we observe *mcp* mRNA is at 6 h.p.i (Fig. 2E), whereas the latest time point is between 18 and 24 h.p.i (Fig. 2H), right before host cell lysis. Interestingly, at these late infection stages, the intracellular location of mRNA varies at different time points. At 6 h.p.i., when the mature VF has been recently established, the *mcp* mRNA is located mainly in a ring surrounding the viral factory (Fig. 2E). However, at 20 h.p.i., the *mcp* mRNA is also situated at discrete sites within the viral factory (Fig. 2H, 3A and Fig. 4B; Fig. S2 and S3), indicating that late Mimivirus *mcp* transcription takes place here. These discrete sites appear as holes within the strong DAPI signal of the VF. Within these holes, the *mcp* mRNA signal is present (Fig. S5), where phase separation (16) is likely responsible for maintaining these sites.

Although the *mcp* mRNA in this study is detectable at the sites of late transcription, previous research has already shown that early transcription starts in the separate viral cores before fusion (9). Therefore, our results suggest that Mimivirus *mcp* mRNA is transported outside the VF at the start of the late infection stage (after fusion of the eVFs) and that upon the progression of the late infection stage, more *mcp* mRNA is produced within the center of the VF. Despite the fusion of the eVFs into one VF, late transcription likely occurs at the same discrete sites where early mRNAs are produced: the remnant sites of the original viral cores.

### The original viral cores in a mature VF may define the location of separate transcription sites

To investigate whether the discrete mRNA spots that we observe at a late infection stage in the mature VF (Fig. 2H) are produced at the remnant sites of the original Mimivirus cores, we followed the course of a Mimivirus infection with an increasing multiplicity of infection (MOI: the number of viral particles per cell).

Consistent with Mutsafi et al., (9), we observe that with an increasing MOI, a higher number of eVFs are formed within each cell (Fig. 3A and B at 4 h.p.i; *Spearman’s rho* = 0.783, *P*-value < 2.2e-16, *S* = 293682, see also Fig. S3). This increase is significant at each MOI step (Fig. 3A and B at 4 h.p.i and Table S2). Since mRNA of the *mcp* gene of Mimivirus is not detectable at this time point, we counted distinct *mcp* mRNA spots at a later stage of the infection, 14 h.p.i. Also, here, we observe that there is a positive correlation between the MOI and the number of distinct viral mRNA spots present in the fused VFs (Fig. 3B at 14 h.p.i; *Spearman’s rho* = 0.749, *P*-value < 2.2e-16, *S* = 260134). The increase in the counted number of mRNA spots is significant at most steps (Fig. 3A and B; Table S2) except for the first (from MOI 0.5 to MOI 2). When comparing the counted eVFs and the distinct mRNA spots, there is no significant difference at most steps, except for MOI 2 (Fig. 3B; Table S2). These results suggest that the original viral cores remain separate transcription sites, even after fusion into one VF. After the fusion of the eVFs, *mcp* mRNA is directly transported outside the VF (Fig. 2E), likely for subsequent translation. Whether this mRNA is produced within the eVFs (between 4 and 6 h.p.i) or in the mature VF right after fusion remains to be investigated.

### More eVFs do not necessarily lead to more viral copy numbers produced by the VF

Together with the increase in MOI, the number of eVFs and the number of mRNA sites, we also observe a positive correlation (Fig. 3C at 4 h.p.i; *Spearman’s rho* = 0.688, *P*-value = 6.428e-11, *S* = 17070) and significant increases in *mcp* DNA copy number at 4 h.p.i up to MOI 5 (Fig. 3C; Table S3), as measured by digital PCR as a proxy for viral copy number. Despite more eVFs being established together with an increase in *mcp* mRNA spots, there is no significant difference in *mcp* DNA copy number between MOI 5 and MOI 10 at 4 h.p.i. This suggests that the maximum space and/or resources for DNA replication are occupied after an MOI of 5 at this intermediate stage of infection. When directly comparing the intermediate and late infection stages, there is a significant increase in DNA copy number at all MOIs (Fig. 3C; Table S3 between 4 and 14 h.p.i). At the late infection stage, the saturation point is reached at MOI 2, as there are no significant differences between MOI 2, MOI 5, and MOI 10 at 14 h.p.i (Fig. 3C; Table S3). Therefore, the differences in MOI mainly affect the DNA copy number at the intermediate stage of infection before the fusion of the eVFs. This observation is backed up by a reported burst of DNA replication during the intermediate infection stage, after the release of the viral genomes into the cytoplasm (9). Once fused into mature VFs, the final output in viral copy number is only affected when the MOI is low, most likely because not all cells in the amoeba population are infected. With a high MOI, when all cells are infected (MOI > 1), it does not appear to matter how many viral cores are delivered to the host cytoplasm; the output in viral copy number remains the same. These results suggest a cap on DNA replication within the mature VF.

### Translation of Mimivirus mRNA takes place in a ring surrounding the VF

Our results demonstrate that exported mRNA remains near the VF (Fig. 2E), suggesting that viral translation might occur here. To investigate this, we have combined smFISH with bioorthogonal non-canonical amino acid tagging (BONCAT). This method allows for the identification of newly synthesized proteins (17–19) and, when combined with fluorescent tags for visualization with fluorescence microscopy, is also referred to as fluorescent non-canonical amino acid tagging (FUNCAT) (20, 21).

Indeed, the mRNA ring outside the VF colocalizes with a ring of FUNCAT signal, suggesting active translation of the viral mRNA at this site (Fig. 4). Recently, we described that giant viruses and their known hosts frequently mismatch in codon usage (22). How giant viruses overcome this mismatch and still ensure efficient viral translation is not clear. For Mimivirus, it seems that the tRNA pool is not significantly altered during the course of infection in their best-known amoeba host (23). This is despite Mimivirus encoding for six tRNA genes. Therefore, we combined smFISH and FUNCAT, with FISH-targeting amoebal ribosomal RNA (rRNA), and we show that the rRNA also colocalizes in a ring surrounding the VF (23; see also Fig. S4), suggesting that Mimivirus manipulates the translation system to overcome the codon usage mismatch.

When labeled with a lipophilic carbocyanine DiD dye, which enhances fluorescence when incorporated into membranes or bound to lipophilic biomolecules such as proteins, we confirmed the presence of these at the same ring surrounding the VF (Fig. S4). This signal likely indicates the host membranes known to be recruited to the VF (10). These host membranes have the characteristic appearance of rough endoplasmic reticulum (ER). They are studded with ribosomes (10), supporting the hypothesis that the translation of Mimivirus mRNA occurs at this ring surrounding the VF.

### Conclusion

With the work presented here, we gained novel insights into giant virus transcription, translation, and DNA replication. Although smFISH has already been used to study active viral infections of a large DNA virus in complex samples (24), it has not yet been used for visualizing the localization of giant virus mRNA. The results in this paper strongly suggest that Mimivirus transcription occurs at the remnant sites of the original viral cores within the fused VF. Recently, it has been shown that the Mimivirus VF is formed by a multilayered phase separation driven by at least two scaffold proteins (16). The presence of proteins associated with transcription in the so-called Inner Layer (a VF compartment analogous to the internal content of the viral core) supports our findings. Moreover, phase separation is likely the driver of maintaining these remnant sites, even after the eVFs are fused into one VF.

Once the Mimivirus cores are delivered within the host cell, early viral transcription starts within these cores. When the viral genome is released from the cores, DNA replication occurs adjacent to these cores/transcription sites (9), which leads to the formation of the eVFs. Our results demonstrate that the number of eVFs present within a single cell does not affect the final output in viral genome copy number. Whether a mature VF can only produce a maximum number of viral genome copies due to space or resource limitations or some sort of regulation remains to be investigated.

We also show that Mimivirus mRNA is transported outside the VF for translation, which occurs at the border of the VF once it is established. Here, mimiviruses differ from poxviruses as, in addition to transcription and DNA replication, translation also occurs within the viral factories for poxviruses (25). The exact mechanism by which Mimivirus mRNA is transported remains to be elucidated. Interestingly, a second Mimivirus VF compartment, the Outer Layer, acts as a selective barrier and recruits VF proteins (16). In a mature VF, the Outer Layer is also localized in a ring around the VF (just like the exported mRNA) and concentrates proteins associated with post-transcriptional regulation. Therefore, we hypothesize that once the mRNA passes the Inner and Outer Layers, it directly encounters the ribosomes on the ER-like host membranes for translation.

Whether the localization of mRNA of other infection stages is the same and whether the localization of transcription and translation is shared among most *Nucleocytoviricota* remains to be illuminated. Exciting is the difference between cytoplasmic and nuclear replication viruses. Although the nuclear viruses transform the host nucleus into a VF, we expect the dynamics to be divergent. Moreover, some *Nucleocytoviricota* encode parts of the translation machinery; therefore, each virus’s genomic information is also likely to have a meaningful impact.

## MATERIALS AND METHODS

### Probe design

Custom Stellaris FISH Probes were designed against the *Acanthamoeba polyphaga mimivirus* (GenBank: HQ336222.2) major capsid protein (*mcp*) gene by utilizing the Stellaris RNA FISH Probe Designer (Biosearch Technologies, Inc., Petaluma, CA) available online at https://www.biosearchtech.com/support/tools/design-software/stellaris-probe-designer (Version 4.2; masking level = 2, oligo length = 20, minimum spacing = 2). To avoid potential off-target binding, we performed blastn (26) searches of the 68 possible probe sequences against the *Acanthamoeba terricola Neff* host genome (RefSeq: GCF_000313135.1) and other giant virus genomes (*Tupanvirus deep ocean*: MF405918.1, *Tupanvirus soda lake*: KY523104.1, *Marseillevirus sp*. strain Vienna: PP736097.1, *Acanthamoeba castellanii medusavirus* J1: AP018495.1). Out of the 68 sequences, we selected 35 probes for smFISH (Table S1). The probe sequences were hybridized with the Quasar 670. Stellaris RNA FISH Probe set labeled with (Biosearch Technologies, Inc.), following the manufacturer’s instructions online at https://www.biosearchtech.com/support/resources/stellaris-rna-fish/stellaris-protocols.

### Sample preparation

*A. terricola* Neff (ATCC 30010) cells were seeded in a 24-well plate (Thermo Fischer Scientific #142475) in Peptone-Yeast Extract-Glucose medium (PYG: ATCC Medium 712) at a concentration of 10^5^ cells/mL, and 1 mL per well. Subsequently, the cells were left to attach at 25°C for 30 min. *Acanthamoeba polyphaga mimivirus* was added at an MOI of 1, and the plates were centrifuged at 1,000 × *g* at room temperature (RT) for 30 min to synchronize the infection. To minimize the possibility of re-infections, the medium was exchanged after centrifugation. For all experiments, the cells were incubated in an incubator at 25°C.

After the desired hours post-infection (h.p.i), the cells were detached by scraping, and the cell suspension of each well (~1 mL) was collected in a 1.5 mL Eppendorf tube. The tubes were centrifuged at 5,000 × *g* at RT for 5 min, 800 µL of the supernatant was removed, and the pellet was resuspended in the remaining 200 µL through vortexing. Then, 50 µL of each cell suspension was added to a well of a microscope slide (Marienfeld #1216690), and the cells were left to attach for 1 h at RT. The supernatant was removed from each well, and the samples were fixed with 20 µL 4% formaldehyde for 10 min at RT. Subsequently, the formaldehyde was removed, and the slides were washed with 40 µL of RNAse-free water. The cells were permeabilized with 40 µL of 70% ethanol for 1 h at 4°C. After permeabilization, 70% ethanol was removed from each well and left to evaporate further. To test whether the probes bind to RNA and not to DNA, an RNase treatment was performed by adding a final concentration of 10 µg/mL RNase A (Thermo Scientific #EN0531) for 30 min at 37°C to the slides after permeabilization.

The number of cells that attach to the microscope slide is higher at the early time points (4 and 6 h.p.i.) compared to the late time points (14 and 20 h.p.i.), as the cells tend to round up at these later time points. Therefore, to harvest a sufficient number of infected cells, we initiate our experiments with a high number of cells and treat them with great care to ensure minimal cell loss before fixation.

### Fluorescent noncanonical amino acid tagging

For performing FUNCAT, the amino acid homopropargylglycine (HPG) was added to the amoeba culture at least 30 min before sampling at a concentration of 50 µM. The dye mix (1.25 µL of 20 mM CuSO_4_, 1.25 µL of 100 mM THPTA, and 0.3 µL 5 mM Cy3-azide) was prepared and incubated in the dark at RT for 3 min. Then, the dye mix was gently mixed with a freshly prepared mix of 221 µL Page’s Amoeba Saline buffer (PAS: ATCC medium 1323), 12.5 µL of 100 mM sodium ascorbate, and 12.5 µL of 100 mM aminoguanidine hydrochloride. Of this final mix, 10 µL was added to each well of the microscope slide. The slides were incubated in the dark at RT for 30 min. Afterward, each well was washed 1–3 times with PAS.

### Single-molecule messenger RNA fluorescence *in situ* hybridization

After permeabilization, and optionally the FUNCAT click reaction, the slide was air-dried and 10 µL of smFISH Hybridization Buffer (24; 20% deionized formamide, 1 mg/mL *E. coli* tRNA, 2× SSC, 0.2 mg/mL BSA, 2 mM ribonucleoside-vanadyl complex, 100 mg/mL dextran sulfate, and nuclease-free water) containing 437.5 nM of probe working solution was added to each well of the slide. For hybridization, the slide was incubated in a moist chamber in the dark at 37°C overnight (~20 h). The smFISH Hybridization Buffer was removed, and the slide was transferred into a 50 mL tube containing Wash Buffer (2× SSC, nuclease-free water) and incubated in a water bath at 39°C for 10 min. After washing, the slides were dipped into ice-cold Milli-Q water and air-dried. The different conditions tested for setting up the smFISH protocol and obtaining an optimal signal are displayed in Table 2.

**TABLE 2:** Conditions tested for performing the smFISH protocol

Step in protocol	Concentrations and/or conditions tested		
	smFISH	rRNA FISH	Stellaris RNA FISH
Sample preparation			
Fixation	4% formaldehyde: 1 h, 30 min, 10 min at RT		
Permeabilization	70% ethanol: 1 h and 30 min at 4°C; proteinase K: 1-10 min at RT		
Hybridization			
Ingredients (final concentration)	1.25, 2.5, 3.75, 4.375, and 5 ng/µL probe mix		
	0%, 10%, 15%, 20%, 25%, 30%, 35%, 40%, and 50% formamide		
	1 mg/mL *E. coli* tRNA	900 mM NaCl	Stellaris hybridization buffer
	2× SSC	20 mM Tris/HCl	
	0.2 mg/mL BSA	0.01% SDS	
	2 mM ribonucleoside-vanadyl complex		
	100 mg/mL dextran sulfate		
	Nuclease-free water		
Temperature	32°C, 37°C, 42°C		
Time	5 h, 17 h, 20 h		
Post-hybridization			
Initial wash	20% formamide, 2× SSC, 15 min at 37°C and 39°C		Wash Buffer A
Final wash	Ice-cold Milli-Q water		Wash Buffer B

### Ribosomal RNA fluorescence *in situ* hybridization

For rRNA FISH, 10 µL of rRNA FISH Hybridization Buffer (900 mM NaCl, 20 mM Tris-HCl pH 8.0, 0.01% SDS, and 25% formamide) containing 5 µM of Euk516 probe (5′-GGAGGGCAAGTCTGGT-3′; labeled with the fluorophore Fluos) working solution was added to each well of the microscopy slide. Hybridization was performed in a moist chamber in the dark at 46°C for 1.5 h. Subsequently, slides were washed with a wash buffer (201 mM Tris-HCl pH 8.0, 50.25 mM EDTA, and 0.149 M NaCl) and kept in the wash buffer in a 50 mL tube at 48°C for 10 min. Then, the slides were dipped in ice-cold Milli-Q water and air-dried.

### DiD staining

DiD (1,1′-dioctadecyl-3,3,3′,3′- tetramethylindodicarbocyanine, 4-chlorobenzenesulfonate salt; Thermo Fischer Scientific #D7757) staining was performed after permeabilization. The staining solution (1 µM DiD in 96-100% Ethanol) was sonicated for 30 min. After this homogenization step, 3 µL of staining solution was added to each well of the microscope slide and incubated at 37°C for 20 min. After incubation, the wells were washed with 40 µL of PAS at RT for 5 min.

### Imaging

After the smFISH, FUNCAT, rRNA FISH, and/or DiD staining, 4,6-diamidin-2-phenylindole (DAPI) (1 µg/mL) was added to the wells on the slides, incubated for 3 min, washed off with nuclease-free water, and air-dried. The slides were embedded in CitiFluor AF1 Mounting Medium (Electron Microscopy Sciences #17970-25) and imaged using a confocal laser-scanning microscope (Leica SP8).

### MOI experiments and digital polymerase chain reaction (dPCR)

*A. terricola* Neff cells were infected with *A. polyphaga mimivirus* (as described above) using an MOI of 0.5, 2, 5, and 10. Each well from a 24-well plate was sampled at 4 and 14 h.p.i., and for each MOI and time point, at least five different replicates (wells) were used, and the experiment was repeated 3 times, giving a minimum of 15 data points per MOI and time point.

For DNA extraction, the content of each well was collected in a 1.5 mL Eppendorf tube and centrifuged for 30 min at 13,000 × *g* at 10°C. The supernatant was replaced with 200 µL PAS, and the pellet was resuspended through vortexing. DNA was extracted using the DNeasy Blood & Tissue Kit (Qiagen #69506), adding 20 µL proteinase K and 200 µL Buffer AL and incubating at 56°C for 30 min at 400 rpm at the first steps. The DNA copy number of Mimivirus was quantified by performing digital PCR (dPCR) on the extracted DNA using the QIAcuity system and QIAcuity EG PCR Kit (Qiagen #250113). Primers targeting the *mcp* gene of Mimivirus (MCP_APolyphagaMimivirus_F:5′-TCGTTTTTACGAAACATGATGG-3′; MCP_ApolyphagaMimivirus_R: 5′-CGATGGTGATTTGGAACACA-3′) were used as a proxy for measuring viral copy number.

For each MOI, the samples were also subjected to smFISH, DAPI staining, and confocal laser-scanning microscopy. To count the number of eVFs at 4 h.p.i, at least 50 infected cells were used per MOI. To count the number of distinct *mcp* mRNA spots at 14 h.p.i, at least 45 infected cells were used per MOI.

### Statistics and data visualization

The data were processed and visualized using R v.4.3.0 (27), with the packages dplyr (28) and ggplot2 (29). Final figures and graphs were made with Inkscape v.1.1.2 (https://inkscape.org).

## Data Availability

The raw microscopy images and data used to generate the figures are available at Zenodo (https://doi.org/10.5281/zenodo.15079574).

## SUPPLEMENTAL MATERIAL

Supplemental figures jvi.00554-25-s0001.pdf Figures S1 to S5.

Supplemental tables jvi.00554-25-s0002.xlsx Tables S1 to S3.

ASM does not own the copyrights to Supplemental Material that may be linked to, or accessed through, an article. The authors have granted ASM a non-exclusive, world-wide license to publish the Supplemental Material files. Please contact the corresponding author directly for reuse.
